# ROSIE, a database of reptilian offspring sex ratios and sex-determining mechanisms, beginning with Testudines

**DOI:** 10.1038/s41597-021-01108-1

**Published:** 2022-01-21

**Authors:** Caleb J. Krueger, Fredric J. Janzen

**Affiliations:** 1 Department of Ecology, Evolution, and Organismal Biology, Iowa State University, Ames, Iowa 50011-3223 USA; 2 Ecology, Evolution, and Behavior Program, W. K. Kellogg Biological Station, Michigan State University, Hickory Corners, MI 49060 USA

**Keywords:** Herpetology, Germline development, Embryology

## Abstract

In contrast to genotypic sex determination (GSD), temperature-dependent sex determination (TSD) in amniotic vertebrates eludes intuitive connections to Fisherian sex-ratio theory. Attempts to draw such connections have driven over 50 years of research on the evolution of sex-determining mechanisms (SDM), perhaps most prominently among species in the order Testudines. Despite regular advancements in our understanding of this topic, no efforts have been published compiling the entirety of data on the relationships between incubation temperature and offspring sex in any taxonomic group. Here, we present the Reptilian Offspring Sex and Incubation Environment (ROSIE) database, a comprehensive set of over 7,000 individual measurements of offspring sex ratios in the order Testudines as well as SDM classifications for 149 species. As the name suggests, we plan to expand the taxonomic coverage of ROSIE to include all non-avian reptiles and will regularly release updates to maintain its comprehensive nature. This resource will enable crucial future research probing the ecology and evolution of SDM, including the presumed sensitivity of TSD to rapid environmental change.

## Abstract (summary)

**Table:** 

Measurement(s)	sex-determining mechanism • offspring sex ratio • sex determination • incubation environment
Technology Type(s)	systematic review study design
Sample Characteristic - Organism	Testudines • Reptile

Machine-accessible metadata file describing the reported data: 10.6084/m9.figshare.16415784

## Background & Summary

Assuming the cost of producing males and females is equal, theory predicts that gonochoristic populations should reach an equilibrium sex ratio of 1:1, a value easily produced at fertilization by the meiotic processes of most forms of genotypic sex determination (GSD)^1,2^. That species therefore would deviate from this sex-determining mechanism (SDM) is remarkable, but deviate they do^3,4^. Perhaps best known among the alternative mechanisms is temperature-dependent sex determination (TSD), where sex is irreversibly determined after fertilization by temperatures experienced in the nest^4–6^, a mechanism that commonly produces single-sex clutches^7–11^. Despite its evident potential to produce non-Fisherian sex ratios, TSD is remarkably widespread among vertebrates; first described in the lizard *Agama agama* by Madeleine Charnier in 1966^12^, it was subsequently confirmed in turtles^13^, fishes^14^, crocodilians^15^, and tuatara^16^. Within these latter two orders, all species display TSD, whereas both GSD and TSD occur in squamates, turtles, and fishes^3,4^. This curious SDM diversity and phylogenetic distribution has spurred a bounty of research over the past 50 + years on the ecology and evolution of SDM, focusing primarily on the mechanism and potential adaptive value of TSD (reviewed by^6,17–21^).

Among taxa in which it is present, the order Testudines has arguably contributed most to our understanding of TSD. Though first described in a lizard, it was the works of Claude Pieau^13^ and Chester Yntema^22^ in *Emys orbicularis* and *Testudo graeca*, and *Chelydra serpentina*, respectively, that brought TSD to the attention of the broader research community. When the community responded by attributing the phenomenon to differential mortality, James Bull and Richard Vogt^23^ confirmed that it was incubation temperature that directly influenced offspring sex in a community of North American turtles; they were also the first to use the term “temperature-dependent sex determination”. Since those pioneering years, chelonian studies have dominated the published literature on TSD, accounting for approximately 50% of all offspring sex-ratio studies in non-avian reptiles despite only comprising ~3% of the species in this group^24^.

Investigations of aspects of the ecology and evolution of TSD in chelonians are published routinely, and the state of our understanding of SDM in reptiles more broadly is regularly summarized every few years^6,17–21^. However, despite the metronomic publication of knowledgeable reviews, limited effort has been made to compile and publish the data from which this knowledge is drawn. In particular, only two efforts have attempted to organize chelonian offspring sex-ratio data, each with their own shortcomings. Paukstis & Janzen^25^ represents the first effort, which includes offspring sex-ratio data spanning the diversity of non-avian reptiles, but only includes results from constant-temperature incubation experiments and can no longer be considered up to date. The more recent compilation^24^ likewise spans non-avian reptiles while also including measurements of additional phenotypes beyond sex that are influenced by incubation temperatures. However, this database excludes studies on natural incubation and exogenous hormone application, two topics often investigated in the ecological/evolutionary literature^18,26–28^. In addition, the authors’ methods largely excluded data outside the scope of *Web of Science* (e.g., unpublished theses/dissertations, select journals).

Such offspring sex-ratio data are necessary to characterize TSD, but a complete understanding of SDM evolution is impossible without comprehensive data on the taxonomic distribution of both TSD and its counterpart GSD as well. Several sources present these data for turtles but suffer from shortcomings much like those described above. For example, the Tree of Sex Database^4^ has, to the best of our knowledge, not been directly updated since its initial release in 2014. In addition, both it and subsequent publications that indirectly expand its taxonomic coverage (e.g.^29^,) have not taken advantage of SDM classifications presented in gray literature, such as publications from conservation breeding programs or unpublished theses/dissertations.

Here, we present the Reptilian Offspring Sex and Incubation Environment (ROSIE) database, a comprehensive compilation of offspring sex ratios and SDM in chelonians, with future plans to include data from all non-avian reptiles. Our database is easily updatable, and can be used to address a variety of key questions, including:

1. What is the ancestral SDM in chelonians, and how often have transitions between mechanisms occurred?
2. How does the relationship between incubation temperature and offspring sex (i.e., the sex-ratio reaction norm) evolve within and among species?
3. To what extent does TSD vary geographically? Temporally? Among species? Within species? Among clutches? Across generations?

## Methods

We obtained hatchling sex-ratio data in turtles using *Web of Science* (v.5.35) to search for research published since the discovery of TSD (1966^12^) until 31 December 2020. On two separate occasions (17 June 2020 and 7 January 2021), we searched all databases for topics including the following terms: sex AND determin* AND incubat*, along with either turtle* or a wildcard version of each species’ taxonomic name to account for suffix variation (e.g., apalon* mutic* for both *Apalone muticus* and *Apalone mutica*). Taxonomy followed the 356 chelonian species identified in Turtle Taxonomy Working Group^30^. We reviewed additional publications and gray literature known to contain hatchling sex-ratio data, as well as research referenced within sources from the systematic search. Altogether, these methods returned 910 sources for evaluation.

**Fig. 1:**
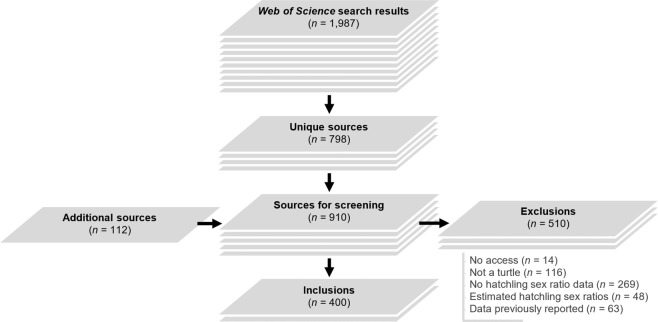
Workflow of literature search and source screening process. *Web of Science* searches on 17 June 2020 and 7 January 2021 returned 798 unique sources for screening, supplemented by 112 sources known to contain offspring sex-ratio data identified outside the *Web of Science* results. After screening, we extracted data from 400 sources for inclusion in the database.

We evaluated sources obtained in the literature search based on the full text, and exclusions fell into the following categories: (1) inaccessible (n = 14), (2) study species was not a turtle (n = 116), (3) hatchling sex ratios were not reported (n = 269), (4) hatchling sex ratios were estimated based on incubation temperatures or durations (n = 48), and (5) hatchling sex-ratio data were previously reported elsewhere (n = 63). After exclusion, 400 sources remained for data extraction (Fig. 1).

From each source, we extracted data on incubation conditions and offspring sex measurements, including additional variables such as hatching success, incubation duration, and sexing methodology (Online-only Table 1)^31^. When variable values (mean incubation duration, sex ratio, etc.) were not provided in the text, tables, or figure legends, we extracted values from figures using WebPlotDigitizer (v4.4^32^). For a number of sources (n = 42), we contacted the corresponding authors to request relevant materials to clarify sample sizes or other questions about the data. We examined all data and exclusions twice to ensure accuracy and, to avoid data replication, we examined data and manuscripts from lab/author groups to determine whether multiple sources analyzed the same information. When sources shared data, we excluded measurements from the more recent source(s) unless (1) additional samples were included, or (2) data were presented in a different format (e.g., sex ratio per shelf in each incubator vs. sex ratio per whole incubator).

We gathered chelonian SDM information in a stepwise manner. First, we compiled classifications from existing databases^4,29^, which were next evaluated for accuracy and supplemented with SDM classifications based on the offspring sex-ratio data collected as described above. Finally, we performed extensive online searches to identify sources supplying SDM classifications for additional species. Where possible for each species, we include relevant citations, SDM classifications, and classification confidence based on a combination of available data, data in closely related species, and author expertise.

**Fig. 2:**
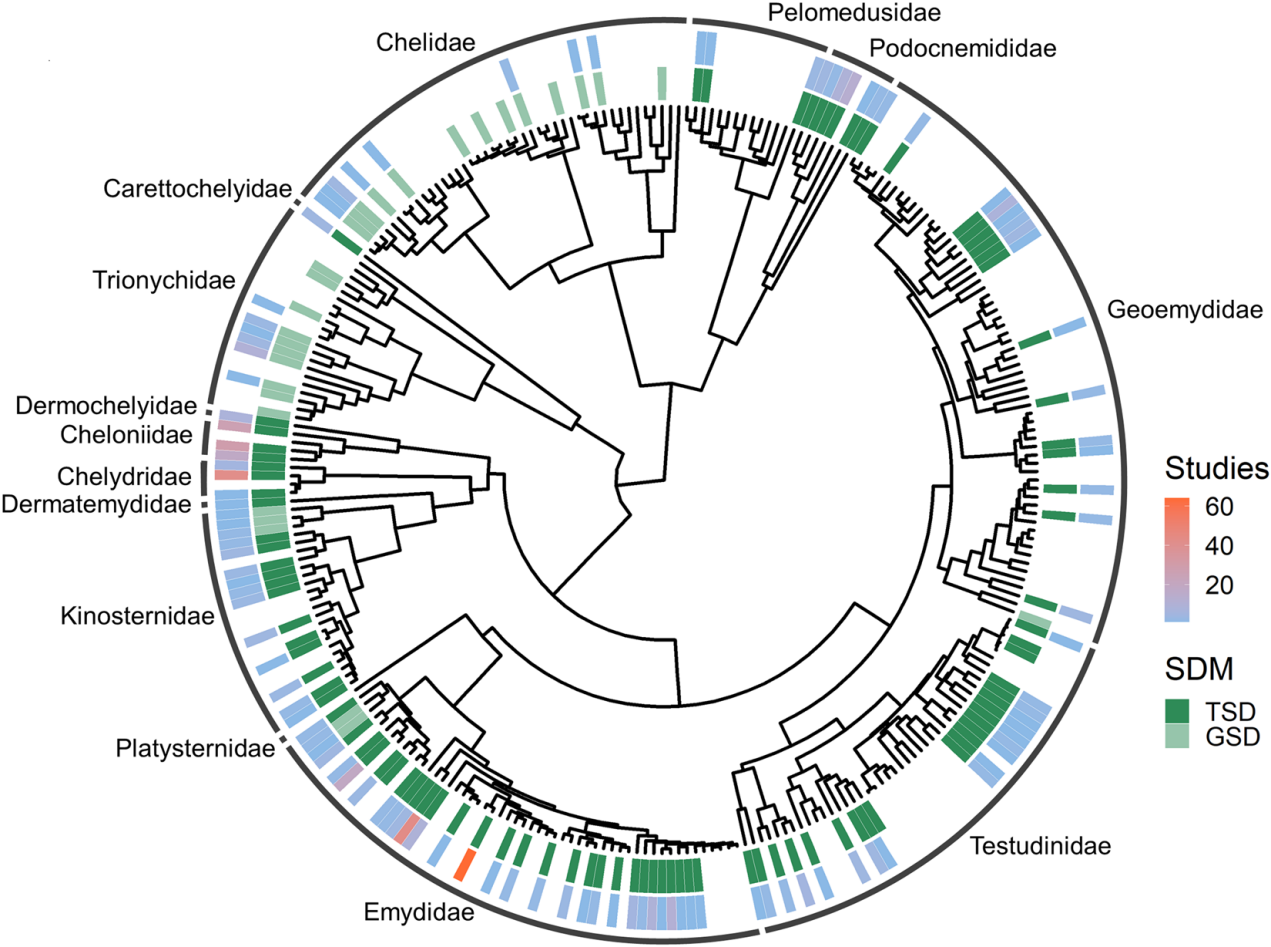
Taxonomic coverage of the database. We downloaded the phylogeny of the order Testudines from a recently published paper^33^, and it covers 279 of 356 turtle species spanning all 14 recognized turtle families (labeled)^30^. The tree includes 111 species found in the offspring sex-ratio database but excludes 6 species that are not represented in the phylogeny (*Batagur affinis*, *Homopus femoralis*, *Lepidochelys kempii*, *Natator depressus*, *Kinosternon alamosae*, and *Podocnemis lewyana*). Additionally, it includes 124 species found in the SDM database but excludes 25 species (*Acanthochelys radiolata*, *Batagur affinis*, *Chelodina oblonga*, *Chelonoidis becki*, *Chelonoidis darwini*, *Chelonoidis donfaustoi*, *Chelonoidis duncanens*, *Chelonoidis guntheri*, *Chelonoidis microphyes*, *Chelonoidis phantasticus*, *Chelonoidis vandenburghi*, *Chelonoidis vicina*, *Eretmochelys imbricata*, *Homopus areolatus*, *Homopus femoralis*, *Kinosternon alamosae*, *Kinosternon stejnegeri*, *Lepidochelys kempii*, *Lissemys ceylonensis*, *Mesoclemmys gibba*, *Natator depressus*, *Nilssonia nigricans*, *Podocnemis lewyana*, *Pseudemydura umbrina*, and *Rhinemys rufipes*).

**Fig. 3:**
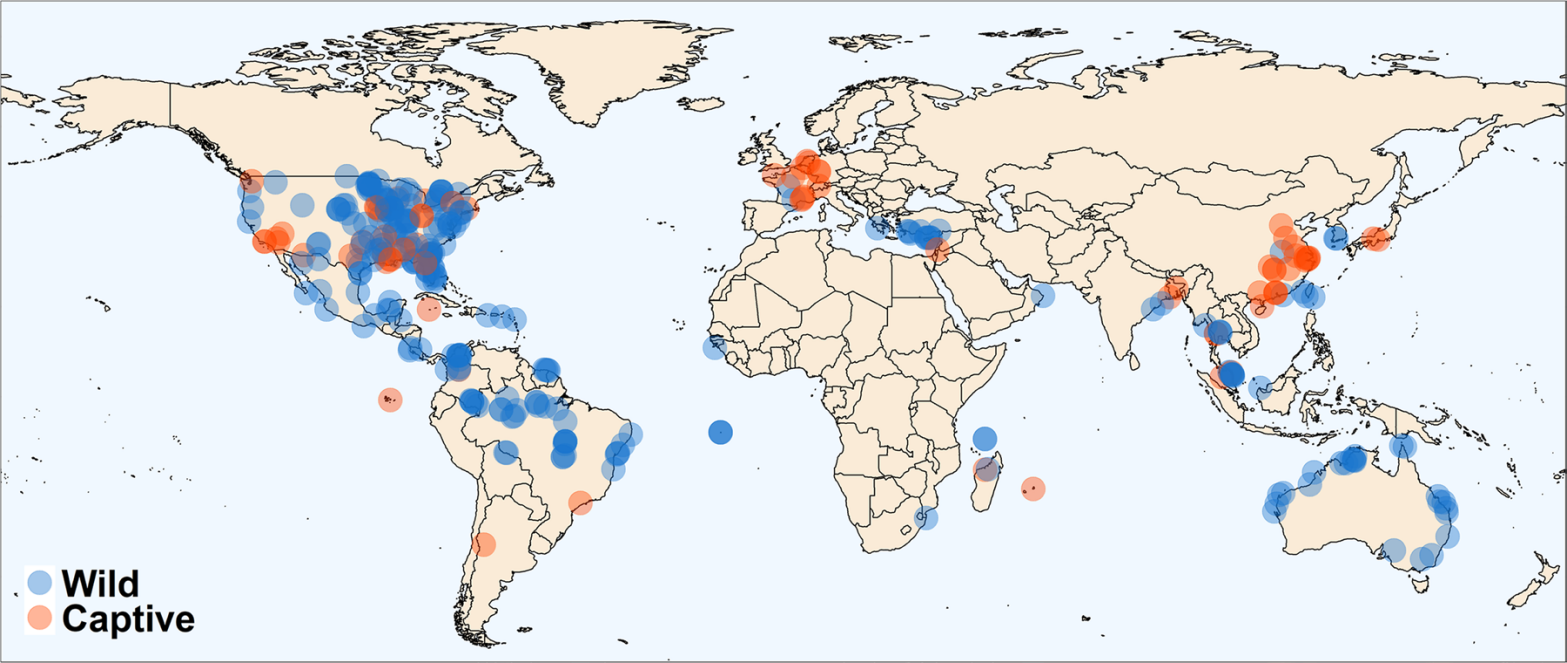
Geographic coverage of the database. The database includes samples from 326 known locations around the globe. 251 represent wild populations (blue points) with the remainder (*n* = 75; red points) representing captively-held populations.

Our offspring sex-ratio compilation contains data on 32.9% (117/356) of recognized chelonian species (Fig. 2), though the taxonomic distribution is highly skewed; just 7 species from 3 families (Chelydridae: *Chelydra serpentina*; Cheloniidae: *Caretta caretta*, *Chelonia mydas*, *Lepidochelys olivacea*; Emydidae: *Trachemys scripta*, *Chrysemys picta*, *Emys orbicularis*) were the focus of nearly half of all studies (45.2%; 241/533; note that some sources contain data on multiple species, hence the difference between total sources [*n* = 400] and total studies [*n* = 533]). The geographic distribution is likewise biased with most studies on wild populations of North American species, starkly contrasting the sampling of African populations (Fig. 3; but note that several African species are represented in captive colonies located outside the continent). Study design is likewise biased, with most (269/400) sources focusing solely or partly on the results of constant-temperature incubation, whereas 85 employ other forms of controlled or semi-controlled incubation conditions (e.g., fluctuating temperatures, temperature switch experiments, room temperature incubation), 124 contain results from natural regimes, and 16 do not define incubation conditions. In addition, 71 studies investigate the influence of chemical applications on offspring sex ratios. In all, the database contains over 7,000 individual measurements of offspring sex ratios, ranging from data on individual eggs to a whole population’s nesting season and representing the sexing of nearly 200,000 turtle hatchlings and embryos.

Our SDM database contains confident SDM classifications for 149 chelonian species (Fig. 2) and unsupported or unlikely classifications for an additional 13 species. Of those with confidently assigned SDM, 24% (36/149) exhibit GSD, 19 of which are also represented in the sex-ratio database (Fig. 2). Besides one species with an unconfident SDM classification (*Chitra chitra*), the remaining species in the sex-ratio database (*n* = 97) comprise a subset of the 113 species confidently known to exhibit TSD. Overall, our collection of chelonian SDM represents a near 50% increase in taxonomic coverage relative to recently published summaries (149 vs 101 species^29^).

As indicated by the name, we plan to expand ROSIE to encompass all non-avian reptile species. In our next update, we will incorporate data from the remaining reptilian orders (Crocodylia, Rhynchocephalia, and Squamata) following the methods described herein, including all data published through the end of 2020. Once ROSIE has reached this final taxonomic scope, we will push updates every other year to include newly available data and maintain the up-to-date nature of this resource.

## Data Records

This database is hosted by GitHub (https://github.com/calebkrueger/ROSIE), and the raw data can be accessed via a unique, stable DOI through Zenodo^31^. The database consists of csv files of (1) extracted offspring sex ratio and incubation environment data with complete references, (2) SDM classifications, (3) excluded sources with complete references and exclusion criteria, and (4) metadata.

## Technical Validation

The data have been thoroughly checked for accuracy by C.J.K. prior to release. The authors urge users to report errors or submit additional data and updates by emailing the corresponding author. Any errors identified can readily be corrected in future updates, which will occur biannually.

## Data Availability

No custom code was used in the creation of this database.

## Online-only Table

**Online-only Table 1:** Values and descriptions of variables extracted for inclusion in the offspring sex-ratio database. .

Column	Value	Description
Order	Text	Order of study organism in reference
Family	Text	Family of study organism in reference
Genus	Text	Genus of study organism in reference
Species	Text	Species of study organism in reference
Subspecies	Text	Subspecies of study organism in reference
Species_Listed_As	Text	Scientific name of study organism provided in reference if not matching current taxonomy
SDM	Text	Confirmed or likely sex-determining mechanism of species
SDM_Confirmed	0	SDM likely
	1	SDM certain
Location	Text	Description of sampling location for eggs or location of incubation if sampling location is not provided
Latitude	Numeric	North-south coordinates for location in decimal degrees
Longitude	Numeric	East-west coordinates for location in decimal degrees
Accurate	0	Organisms sampled over a large area (e.g., multiple states/provinces) or description of location is vague (e.g., “Southeast United States”)
	0.5	Organisms sampled over an intermediately-sized area (state county, several close water bodies, etc.)
	1	Organisms sampled in a small area, well-described location
Captive	0	Eggs taken from wild individuals
	0.5	Eggs taken from both captive and wild individuals
	1	Eggs taken from captive individuals; includes wild-caught and captive-raised
Outdoors	0	Nesting adults live indoors
	0.5	Nesting adults live indoors and outdoors
	1	Nesting adults live outdoors
Within_Species_Range	0	Nesting adults live within native range of the species
	1	Nesting adults live outside native range of the species
Nest_Year_Start	Integer	Start of turtle nesting / egg collection
Nest_Year_Finish	Integer	End of turtle nesting / egg collection
Nest_Month_Start	Integer	Start of turtle nesting / egg collection
Nest_Month_Finish	Integer	End of turtle nesting / egg collection
Incubation_Setup	Artificial nest	Eggs incubated in a human-made nest
	Backswitch	Eggs switched between incubation temperatures at select points in development
	Constant	Eggs incubated at single, unchanging temperature throughout development
	Fluctuating	Eggs incubated under temperatures varying a set amount around a mean value
	Indoor container	Eggs incubated in a container indoors; does not necessarily indicate controlled incubation temperatures
	Natural program	Eggs incubated in a programmable incubator set to match a natural temperature regime
	Nest	Eggs incubated in natural nest
	Outdoor container	Eggs incubated in a container outdoors, generally under an open shelter (i.e., pavilion, covered patio)
Substrate	Text	Description of incubation substrate; may also include information on ratio of substrate:water
Water_Content	Negative integer	Water potential in units of kPa
	Percentage	Percent soil moisture
Humidity	Numeric	Relative humidity of incubation chamber between 0 and 1
Thermometer_Location	Container	Temperature recorded within the incubation container
	Container center	Temperature recorded in the center of the eggs within the incubation container
	Incubator	Temperature recorded within the incubator
	Incubator center	Temperature recorded in the center of the incubator
	Incubator shelf	Temperature recorded on each shelf in the incubator
	Nest	Temperature recorded in the nest
	Nest adjacent	Temperature recorded in substrate directly adjacent to the nest
	Nest bottom	Temperature recorded at the bottom of the nest below all eggs
	Nest center	Temperature recorded in the center of the eggs in the nest
	Nest top	Temperature recorded at the top of the nest above all eggs
	Next to each egg	Temperature recorded by each egg within the incubator
Set_Temp	Numeric	Incubator temperature setting in degrees Celsius
Mean_Temp	Numeric	Mean of actual/recorded incubator temperature in degrees Celsius
CTE	Numeric	Constant temperature equivalent (CTE) for variable incubation regimes in degrees Celsius
Fluctuation	Numeric	Fluctuation above and below mean temperature in degrees Celsius
Mean_Temp_SE	Numeric	Standard error of mean temperature value in degrees Celsius
Mean_Temp_SD	Numeric	Standard deviation of mean temperature value in degrees Celsius
Min_Temp	Numeric	Minimum temperature experienced during incubation in degrees Celsius
Max_Temp	Numeric	Maximum temperature experienced during incubation in degrees Celsius
TSP_Mean_Temp	Numeric	Mean temperature during the thermosensitive period of development (TSP) in degrees Celsius
TSP_Mean_SE	Numeric	Standard error of mean temperature during TSP in degrees Celsius
TSP_Mean_SD	Numeric	Standard deviation of mean temperature during TSP in degrees Celsius
TSP_Variance	Numeric	Variance of mean temperature during TSP in degrees Celsius
TSP_Min_Temp	Numeric	Minimum temperature experienced during the TSP in degrees Celsius
TSP_Max_Temp	Numeric	Maximum temperature experienced during the TSP in degrees Celsius
Backswitch_Start	Integer	Day at which eggs are switched to second incubation temperature
	Text	Developmental stage at which eggs are switched to second incubation temperature
Backswitch_Length	Integer	Length of time spent at second incubation temperature in days
	Text	Developmental stage at which eggs leave the second incubation temperature
Backswitch_Base_Temp	Numeric	Incubation temperature in degrees Celsius prior to first switch
Backswitch_Temp	Numeric	Incubation temperature in degrees Celsius after first switch
Backswitch_Final_Temp	Numeric	Incubation temperature in degrees Celsius after second switch
Chemical_Treatment	Text	Name of chemical applied during embryonic development
Chemical_Mass_μg	Numeric	Mass of chemical applied to egg in micrograms
Chemical_Concentration	Numeric	Molar concentration of chemical solution applied to egg if liquid, % concentration for gases and ethanol given as a value between 0 and 1
Chemical_Volume_μL	Numeric	Volume of chemical solution applied to egg in microliters
Number_Applications	Integer	Number of times the chemical treatment was applied
	Daily	Treatment applied every day of incubation
	Constant	Eggs exposed to gaseous treatment continually
Application_Route	Gas	Gas concentrations (CO2, O2) manipulated in incubation chambers
	Injection	Chemical treatment injected into developing egg
	Substrate	Chemical treatment mixed with incubation substrate
	Topical	Chemical treatment applied to egg surface
Stage_First_Application	Numeric	Developmental stage at which the chemical treatment was first applied
Day_First_Application	Integer	Days since beginning of incubation at which the chemical treatment was first applied
Stage_Last_Application	Integer	Developmental stage at which the chemical treatment was last applied
Day_Last_Application	Integer	Days since beginning of incubation at which the chemical treatment was last applied
Number_Clutches_Experiment	Integer	Total number of clutches used in reference
Number_Clutches_Treatment	Integer	Number of clutches used in specific data row
Clutch_ID	Integer	Unique numbers of each clutch used in specific data row
Total_Incubated_Experiment	Integer	Total number of eggs used in reference
Total_Incubated_Treatment	Integer	Number of eggs used in specific data row
Total_Hatched	Integer	Number of hatchlings produced
Proportion_Hatching	Numeric	Proportion of eggs producing hatchlings
Mean_Incubation_Duration	Numeric	Mean time in days between beginning of incubation and hatching
Incubation_Standard_Error	Numeric	Standard error of mean incubation duration
Incubation_Standard_Deviation	Numeric	Standard deviation of mean incubation duration
Min_Incubation_Duration	Numeric	Shortest incubation duration in days
Max_Incubation_Duration	Numeric	Longest incubation duration in days
Sexing_Method	Endoscopy	Determined sex via endoscopic examination of gonads
	Histology	Determined sex via microscopic examination of gonad slides
	Hormone assay	Determined sex via radioimmunoassays of relevant hormones
	Macroscopy	Determined sex via macroscopic examination of gonad structure
	Morphometrics	Determined sex via external morphometry (e.g., shell shape)
	RT-qPCR	Determined sex via relative expression of sex-linked genes
	Secondary characteristics	Determined sex via sexually dimorphic traits
Sexed_Condition	Dead	Sexed offspring that died naturally
	Live	Non-lethal sexing of live offspring
	Sacrificed	Lethal sexing of live offspring
Sexed_Age_Class	Adults	Sexed mature adults raised in captivity from known incubation conditions
	Embryos	Sexed embryos prior to completion of development
	Hatchlings	Sexed individuals < 1 year after hatching
	Juveniles	Sexed individuals > 1 year old but not yet sexually mature
Sexed_Age_Weeks	Numeric	Time in weeks between hatching and sexing procedure; indicates youngest age sexed if multiple ages sexed
	Text	Embryonic stage at which sexing procedure occurred; indicates earliest stage sexed if multiple stages sexed
Vouchers	0.5	Some voucher specimens deposited in collection
	1	All specimens deposited in collection
Total_Sexed	Numeric	Total number of individuals sexed
Males	Numeric	Number of individuals presenting testes
Females	Numeric	Number of individuals presenting ovaries
Intersex	Numeric	Number of individuals presenting intermediate gonads
Undetermined	Numeric	Number of individuals unable to be sexed accurately (e.g., due to damage)
Proportion_Male	Numeric	Proportion of sexed individuals presenting testes
Proportion_Female	Numeric	Proportion of sexed individuals presenting ovaries
Source	Text	Full citation of included reference in English or original language if no English version is available
Source_Alternate	Text	Full citation of included reference in a second language
WoS	Integer	Number of times reference occurred in Web of Science search results
Other	0	Reference found in Web of Science search results
	1	Reference found outside of Web of Science search results
Data_Location	Text	Description of data location within reference (text, figures, tables, supplements, etc.)
Data_Elsewhere	0	Data is not duplicated in any other reference within the database
	1	Data represents a subset of data in another reference or the same data presented in a different format (e.g., sex ratios broken down by clutch vs by study year)
	?	Data is thought to represent a subset of data in another reference or the same data presented in a different format
Notes	Text	Additional notes on details in reference, data entered in database, etc.

Extracted values are found in “Database.csv”.
